# Probing of Interactions of Magnetite Nanoparticles Coated with Native and Aminated Starch with a DPPC Model Membrane

**DOI:** 10.3390/ijms22115939

**Published:** 2021-05-31

**Authors:** Emilia Piosik, Aleksandra Zaryczniak, Kinga Mylkie, Marta Ziegler-Borowska

**Affiliations:** 1 Faculty of Material Engineering and Technical Physics, Poznan University of Technology, Piotrowo 3, 60-965 Poznan, Poland; aleksandra.zaryczniak@student.put.poznan.pl; 2 Faculty of Chemistry, Nicolaus Copernicus University in Torun, Gagarina 7, 87-100 Torun, Poland; kinga.mylkie@o2.pl

**Keywords:** magnetite nanoparticles, starch, aminated starch, cell membrane, Langmuir film, nanomedicine

## Abstract

Understanding the mechanism of interactions between magnetite nanoparticles and phospholipids that form cellular membranes at the molecular level is of crucial importance for their safe and effective application in medicine (e.g., magnetic resonance imaging, targeted drug delivery, and hyperthermia-based anticancer therapy). In these interactions, their surface coating plays a crucial role because even a small modification to its structure can cause significant changes to the behaviour of the magnetite nanoparticles that come in contact with a biomembrane. In this work, the influence of the magnetite nanoparticles functionalized with native and aminated starch on the thermodynamics, morphology, and dilatational elasticity of the model cell membranes was studied. The model cell membranes constituted the Langmuir monolayers formed at the air–water interface of dipalmitoylphosphatidylcholine (DPPC). The surface of the aminated starch-coated nanoparticles was enriched in highly reactive amino groups, which allowed more effective binding of drugs and biomolecules suitable for specific nano–bio applications. The studies indicated that the presence of these groups also reduced to some extent the disruptive effect of the magnetite nanoparticles on the model membranes and improved their adsorption.

## 1. Introduction

Magnetite nanoparticles (Fe_3_O_4_) show application potential in magnetic resonance imaging, targeted drug delivery, bacteria detection, cell labelling, bioseparation, and anticancer therapy based on hyperthermia [1,2,3,4,5,6,7,8,9,10,11]. Numerous possible applications of magnetite nanoparticles in nanomedicine result from their unique physicochemical properties such as small size, wide chemical affinity, and superparamagnetic properties. Their size is smaller than, or comparable to, that of a cell (10–100 μm), a virus (20–40 nm), a bacterium (0.5–5 μm), a protein (5–50 nm) or a gene (2 × 10–100 nm), which enables them to operate at the biomolecular level effectively. The wide chemical affinity of the magnetite nanoparticles allows the tailoring of their surfaces to adjust to their characteristics to specific medical applications, e.g., they can be coated with drugs or biomolecules to make them suitable for interacting with, or binding to, a desired biological entity [12,13,14,15,16,17]. Moreover, magnetite nanoparticles with superparamagnetic properties upon exposure to an external magnetic field exhibit the ability to be magnetized, but no permanent magnetization remains after the field is turned off [18]. Because of the absence of magnetic remanence, in vivo aggregation of nanoparticles can be avoided. Other advantages of magnetite nanoparticles in nanomedicine are the possibility of controlling their transport by an external magnetic field gradient and their ability to generate heat in the presence of an alternating high-frequency magnetic field. They can accumulate in a desired place in a human body by the use of an external magnetic field, which is important for targeted drug and gene delivery [19]. Their heat generating ability in an alternating magnetic field also allows heat-triggered drug release, which can be applied in hyperthermia to damage cancer cells irreversibly by raising their temperature abnormally [20].

The successful application of magnetite nanoparticles in nanomedicine requires modification of their surface to prevent oxidation and self-association [15]. Their tendency for self-association leads to thermodynamically and magnetically induced aggregation and agglomeration, which negatively affects the unique magnetic properties essential for nanomedicine. Naked magnetite nanoparticles show also insufficient biocompatibility and biodegradability as well as chemical instability under physiological conditions [6], but these obstacles can be overcome by functionalization with natural polymers, which provides excellent colloidal stability and optimal bioperformance. Polysaccharides such as dextran, chitosan, and starch, belong to the group of the most widely studied natural polymers for coating magnetite nanoparticles, a modification that results in the surface presence of highly reactive amino, carboxyl and hydroxyl functional groups [6]. It makes further chemical modification easy and allows for the binding of bioactive substances and bioligands (i.e., drugs, proteins, enzymes, antibodies or DNA) for nano–bio applications.

Magnetite nanoparticles interact with targeted and various non-targeted cells, extracellular matrices, blood plasma and the body’s immune system. In the past decade, numerous types of these nanoparticles with different coatings have been synthesised and characterised. However, because understanding of the interactions between magnetite nanoparticles and biological cells is still limited, only a few nanomedicines based on them have been brought onto the commercial market [21]. In these interactions, the surface coating plays a crucial role because a small modification to its structure can cause significant changes to the behaviour of the nanoparticles when they come in contact with a biomembrane, the first barrier encountered in a physiological environment. A biomembrane is a dynamic complex of a phospholipid bilayer within which (or on its surface) are proteins (receptors, ion channels, glycoproteins), cholesterol, and polysaccharides. For this reason, it is beneficial to employ simplified biomimetic membrane models of known composition such as lipid vesicles (liposomes) and planar lipid layers (Langmuir monolayers, black lipid membranes, freely suspended layers, supported lipid films) to study magnetite nanoparticle–biomembrane interactions [22,23]. The Langmuir phospholipid monolayers belong to the most frequently used cell membrane models [24,25,26,27,28,29,30] and can be formed at the air–water interface of one phospholipid type and from multicomponent mixtures of phospholipids, cholesterol, or proteins. Studies involving the one-component Langmuir films are beneficial because the interactions between the nanoparticles and individual components of the biomembrane can be assessed. Such knowledge is useful during further research using more complex biomembrane models and whole cells. Moreover, external experimental conditions such as temperature and pH can be controlled using the Langmuir method, which enables good reproduction of conditions in different regions of the body. This method, contrary to the vesicle model, provides qualitative information on the influence of the nanoparticles on the phase state, fluidity, and morphology of the model biomembranes. The Langmuir technique undoubtedly allows the establishment of good in vitro models of various kinds of healthy and cancerous cells. However, the most important advantage of this method is the possibility of investigating not only a cell’s overall response but also a mechanism of nano–bio interactions at the molecular level. The Langmuir monolayers were extensively used as cell membrane models in studies on the interactions between different kinds of nanoparticles and phospholipids [31,32,33,34,35,36,37,38].

In this work, interactions of magnetite nanoparticles coated with native starch (Fe_3_O_4_–S) and aminated starch (Fe_3_O_4_–AS) that showed application potential in nanomedicine with a model biological membrane were studied. The surface of the Fe_3_O_4_–AS nanoparticles was enriched in highly reactive amino groups, which allowed more effective binding of drugs and biomolecules suitable for specific nano–bio applications. The Langmuir monolayer formed by dipalmitoylphosphatidylcholine (DPPC) at the air–water interface constituted the model cellular membrane. The research delivered information on the influence of Fe_3_O_4_–S and Fe_3_O_4_–AS nanoparticles on the thermodynamic state, phase transitions, morphology, and dilatational elasticity of the DPPC monolayer. The adsorption process of the investigated nanoparticles into the phospholipid Langmuir film was also studied. As a result, the effect of the amination of the starch in a shell of magnetite nanoparticles on their interactions with DPPC molecules was evaluated at the molecular level.

## 2. Results and Discussion

### 2.1. Surface Pressure-Mean Molecular Area per DPPC Molecule Isotherms

**Figure 1:**
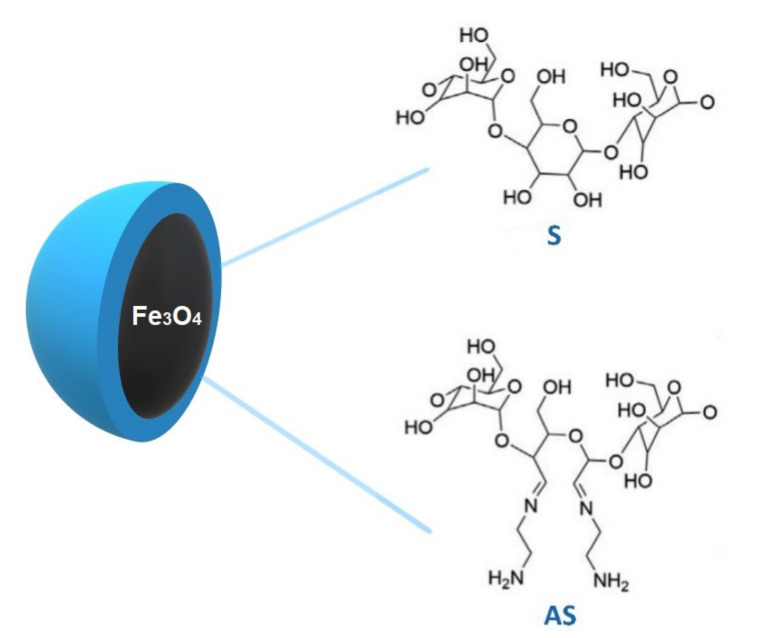
Structure of the magnetite nanoparticles coated with native starch (Fe_3_O_4_–S) and aminated starch (Fe_3_O_4_–AS).

**Figure 2:**
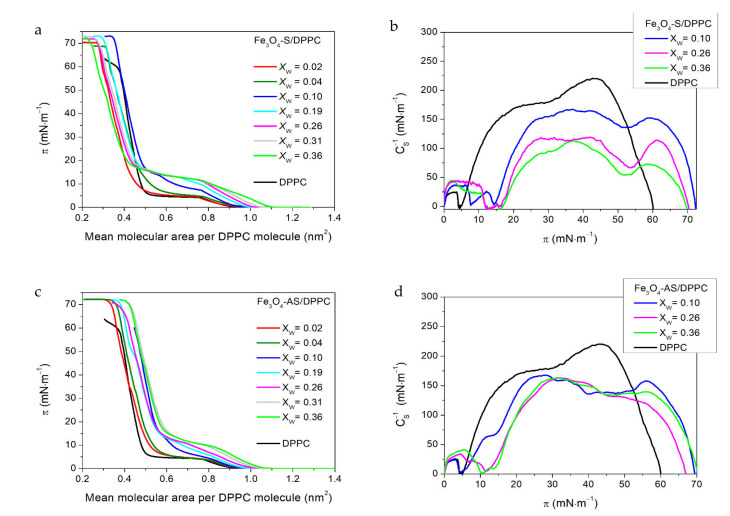
Compression isotherms and compression modulus-surface pressure dependences obtained for the Fe_3_O_4_–S/DPPC (**a**,**b**) and Fe_3_O_4_–AS/DPPC (**c**,**d**) Langmuir monolayers.

To study the influence of the Fe_3_O_4_–S and Fe_3_O_4_–AS nanoparticles (Figure 1) on the thermodynamic behaviour of the DPPC monolayers, mixtures of the investigated nanoparticles and phospholipids were compressed at the air–water interface. The surface pressure (*π*) versus the mean molecular area per DPPC molecule (*A*) isotherms (*π*–*A* isotherms) recorded during their compression are presented in Figure 2a,c. The films formed by the Fe_3_O_4_–S and Fe_3_O_4_–AS nanoparticles and DPPC are denoted by Fe_3_O_4_–S/DPPC and Fe_3_O_4_–AS/DPPC, respectively. *X*_W_ is a weight fraction of the nanoparticles in the mixture that were used to form the binary films. In the plots in Figure 2, the *π*–*A* isotherm recorded during the compression of pure DPPC is also shown for comparison (black lines). The isotherm of DPPC is consistent with those presented in the literature [39,40,41].

The compression modulus (*C*_S_^−1^) versus surface pressure dependencies (*C*_S_^−1^-*π*) plotted in Figure 2b,d provided detailed insight into the phase state of the compressed binary Langmuir monolayers. The *C*_S_^−1^ values were calculated using the following equation found by differentiating the data obtained from the *π*–*A* isotherms.

*C_S_*^−1^ = −*A* δπ/δ*A* (1)

The phase state of the Langmuir films was classified according to the criterion given by Davies and Rideal [42]. According to this criterion, *C*_S_^−1^ ranges from 12.5 to 50 mN·m^−1^ for a liquid-expanded (LE) phase, 50 to 100 mN·m^−1^ for a liquid state (L), 100 to 250 mN·m^−1^ for a liquid-condensed (LC) phase, and more than 250 mN·m^−1^ for a solid state (S). The minima in the plots of the *C*_S_^−1^ versus *π* dependencies (*C*_S_^−1^-*π*) indicate the surface pressures at which a phase transitions or significant reorganization of molecules occurs. *C*_S_^−1^, as the reciprocal of the compressibility, is also a rheological quantity related to the quasi-equilibrium dilatational elasticity of the Langmuir films, that delivers information on their rigidity and capability to store elastic energy.

The Fe_3_O_4_–S and Fe_3_O_4_–AS nanoparticles do not create stable one-component Langmuir films due to their hydrophilic nature. Some of them sink in the water subphase during compression, which makes recorded isotherms unrepeatable. However, the binary monolayers formed from both types of nanoparticles and DPPC are compressible up to the particular concentration of the nanoparticles in a compressed mixture. Their concentration in the mixtures used for the Langmuir films’ formation increased as long as the recorded *π*–*A* isotherms were repeatable. The course of the compression isotherms for the two-component films differed from that of the DPPC isotherm for monolayers formed from mixtures containing the Fe_3_O_4_–S and Fe_3_O_4_–AS nanoparticles. The isotherms recorded for the mixed films are less steep (Figure 2a,c). The stability of the binary films was good enough to reproduce the compression isotherms, but they were less stable than the DPPC monolayer. Moreover, the compression isotherms indicated a smaller stability for the Fe_3_O_4_–S films compared to the Fe_3_O_4_–AS ones.

The isotherms of the mixed films shifted towards the greater areas per a DPPC molecule at the beginning of the compression stage. This shift increased with the concentration of the nanoparticles and was reflected in a value change for an extrapolated mean molecular area (*A*_EXT_) that corresponded to the phase transition of the monolayers to the LE phase. *A*_EXT_ is determined by the extrapolation of the tangent of a first linear slope of the isotherms to *π* = 0. The *A*_EXT_ increase is equal to 0.19 nm^2^ and 0.14 nm^2^ for films with the highest concentration of nanoparticles among these investigated: Fe_3_O_4_–S/DPPC and Fe_3_O_4_–AS/DPPC monolayers with X_W_ = 0.36, respectively. The monolayer expansion confirms the presence of nanoparticles at the air–water interface at the beginning of the compression stage. However, it is worth noticing that after the phase transition from the LE state to the liquid condensed (LC) phase, the isotherms recorded for Fe_3_O_4_–S/DPPC films in the region of the high surface pressure values shifted to the smaller mean molecular areas in comparison to the isotherm for DPPC, while those obtained for Fe_3_O_4_–AS/DPPC monolayers remain shifted to the greater mean molecular areas. The Fe_3_O_4_–S nanoparticles were thus probably squeezing out of the film upon compression. Moreover, part of the DPPC molecules was very likely removed from the air–water interface together with them. On the other hand, the Fe_3_O_4_–AS nanoparticles seemed to remain in the binary films until the end of the compression. Matshaya et al*.,* during studies on the influence of magnetite nanoparticles modified with oleic acid (Fe_3_O_4_–OA) on the phospholipid Langmuir monolayers, observed their distinctive interactions with the saturated and unsaturated phospholipids [37]. The research of Matshaya et al. showed that the interaction between Fe_3_O_4_–OA nanoparticles and saturated phospholipids such as DPPC in binary monolayers caused the shift of their compression isotherms to much smaller mean molecular areas in comparison to those recorded for one-component films formed by nanoparticles and phospholipids. The research authors ascribed this effect to the loss of part of the material from the air–water interface. On the other hand, the presence of the Fe_3_O_4_–OA nanoparticles in the binary films they formed, together with unsaturated phospholipids, led to the shift of compression isotherms to much greater mean molecular areas than in the case of pure phospholipid monolayers and simultaneously much smaller than in the case of the film of the nanoparticles. The difference in the interaction of magnetite nanoparticles with DPPC in the current work and those studied by Matshaya et al. highlights the important role played by the coating in nano–bio relations.

The phase transition of the DPPC monolayer from the LE state to the LC phase is represented in the *π*–*A* isotherm by an almost horizontal plateau starting at *A*_C_ = 0.77 nm^2^ and *π*_C_ ≈ 5 mN·m^−1^. The introduction of the Fe_3_O_4_–S and Fe_3_O_4_–AS nanoparticles into the DPPC film makes the plateau region no longer horizontal. Moreover, the increase in the concentration of both types of nanoparticles in the DPPC monolayer led first to a shortening of the plateau region and then to its almost total disappearance. The loss of plateau horizontality caused the occurrence of two inflection points associated with the transition from LE state to LC phase in the isotherms recorded for the binary films starting from the Fe_3_O_4_–S/DPPC and Fe_3_O_4_–AS/DPPC monolayers with X_W_ = 0.10. The first one appeared at the lower surface pressure *π*’_C_ and was related to the beginning of this phase transition, while the second one emerged at the higher surface pressure *π*”_C_ and corresponded to its end: *π*’_C_ and *π*”_C_ take higher and higher values when the concentration of nanoparticles in the mixed films grows. The presence of the Fe_3_O_4_–S nanoparticles in the binary films modified the parameters of the LE–LC phase transition much more than the Fe_3_O_4_–AS ones did. For instance, the *π*’_C_ value increased in the case of the Fe_3_O_4_–S/DPPC monolayer with X_W_ = 0.36 up to *π*’_C_ ≈ 12 mN·m^−1^, while for the Fe_3_O_4_–AS/DPPC film with X_W_ = 0.36 it increased up to *π*’_C_ ≈ 10 mN·m^−1^.

Figure 2b,d show that the Fe_3_O_4_–S and Fe_3_O_4_–AS nanoparticles present in the DPPC monolayer modified its elasticity. In the plots of the *C*_S_^−1^-*π* dependencies obtained for the DPPC monolayer and binary films, the main maxima corresponding to the LE and LC phases can be distinguished. The maximal value of *C*_S_^−1^ determined for the binary films in the LE phase was higher than in the case of the pure DPPC monolayer in the same phase state. On the other hand, a decrease in the maximal *C*_S_^−1^ of the mixed films in the LC phase was observed. It could clearly be seen that the presence of the nanoparticles in the DPPC monolayer caused the increase in the lateral packing density of the phospholipid molecules at the beginning of the compression stage when the monolayer was in the LE state. Further compression of the binary films to the surface pressure above the LE–LC phase transition resulted in the decrease of stability and lateral packing density in comparison to the pure DPPC monolayer. One could also see (Figure 2b,d) that this decrease was much stronger for films containing the Fe_3_O_4_–S nanoparticles than for those formed by DPPC with the Fe_3_O_4_–AS nanoparticles. The effect could be related to squeezing a part of the nanoparticles, together with some phospholipid molecules, out of the Langmuir monolayers upon compression, which was stronger in the case of the binary films containing the Fe_3_O_4_–S nanoparticles as postulated based on the analysis of the *π*–*A* isotherms.

### 2.2. Brewster Angle Images

**Figure 3:**
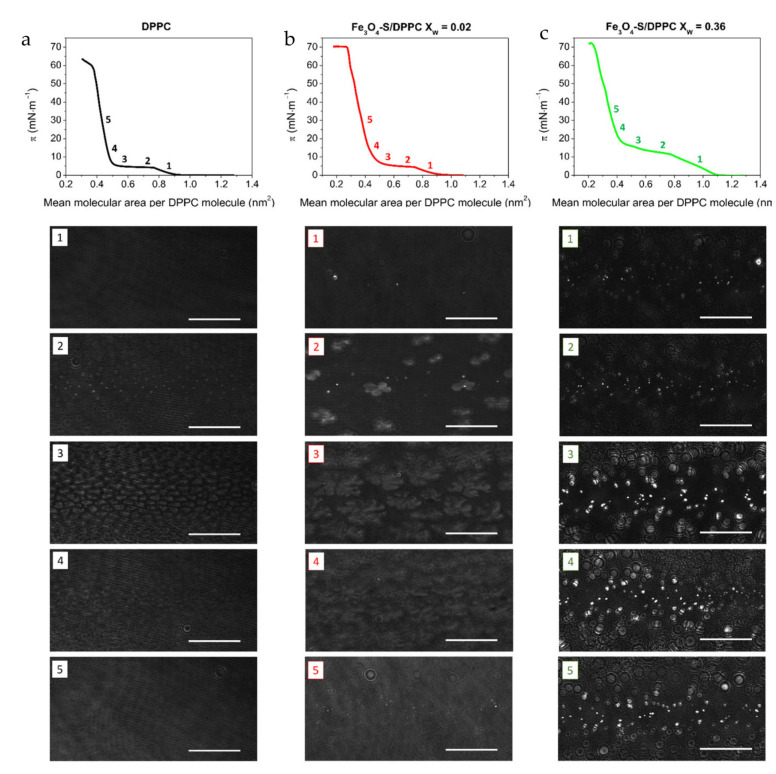
Brewster angle microscope images recorded during compression of the DPPC monolayer (**a**) and Fe_3_O_4_–S/DPPC films with *X*_W_ = 0.02 (**b**) and *X*_W_ = 0.36 (**c**). Length of scale bar in BAM images—100 µm.

A Brewster angle microscope (BAM) is a useful tool for assessing the effect of magnetite nanoparticles on the morphology of model biomembranes formed by the Langmuir technique. The BAM images recorded during compression of the DPPC and Fe_3_O_4_–S/DPPC films with different concentrations of nanoparticles are shown in Figure 3. Numbers placed next to the *π*–*A* isotherms correspond to the compression stages, during which the BAM images (shown below the isotherms) were taken. The Fe_3_O_4_–AS nanoparticles influenced the morphology of the DPPC monolayer similarly to the Fe_3_O_4_–S nanoparticles. For this reason, the BAM images recorded for the Fe_3_O_4_–AS/DPPC monolayers are shown in Supplementary Materials Figure S1.

The BAM images taken during the first increase in the surface pressure taking place upon compression of the pure DPPC, when the phospholipid monolayer was in the liquid-expanded phase, revealed a homogeneous texture (Figure 3a). In the case of the binary films at this compression stage, the formation of small circular domains was observed (Figure 3b,c). The number of the domains grew in tandem with the concentration of the Fe_3_O_4_–S nanoparticles, which indicated that they were probably created by the nanoparticles under investigation. The formation of the micrometric domains by the magnetite nanoparticles has a negative effect because it can lead to the vanishing of the superparamagnetic properties, feature resulting from their nanometric size. This negative effect was observed also for other hydrophilic [43] as well as hydrophobic [37] magnetite nanoparticles in the binary monolayers formed with saturated and unsaturated phospholipids. At the plateau region, the coexistence of the LE and LC phases occurred in the case of the pure DPPC film. In the BAM images, at this compression stage, the phospholipid LC domains are visible (Figure 3a). The dark background corresponds to the DPPC monolayer in the LE state. Further compression caused an increase in the size of the DPPC LC domains, and finally the formation of a homogenous monolayer in the LC phase when the plateau ended. The BAM images obtained for the Fe_3_O_4_–S/DPPC film with X_W_ = 0.02 at the plateau region revealed the presence of the small circular domains of the Fe_3_O_4_–S nanoparticles between the LC phospholipid domains. The tendency to preferentially partition the LE phase of the DPPC monolayer also applies to the magnetite nanoparticles coated with oleic acid and aminated chitosan studied, respectively, by Matshaya et al. [37] and Piosik et al. [43]. The formation of the separate domains by the Fe_3_O_4_–S nanoparticles and DPPC molecules indicated the partial or total immiscibility of the components in the binary films. Incomplete miscibility of the components in the mixed films seemed to be characteristic of the systems formed by the magnetite nanoparticles with different coatings and phospholipids from the group of the phosphatidylcholines [37,43]. It is worth noting that the size of the DPPC domains formed in the binary Fe_3_O_4_–S/DPPC film with X_W_ = 0.02 was significantly larger than those created in the one-component monolayer at a particular compression stage. The larger size of the DPPC domains in the two-component films suggested that the neighborhood of other phospholipid molecules was more energetically beneficial for DPPC than in the vicinity of the nanoparticles. After the plateau ended, the Fe_3_O_4_–S domains with the phospholipid monolayer in the LC state as the background were observed in the BAM images recorded for the Fe_3_O_4_–S/DPPC film with X_W_ = 0.02. In the case of the Fe_3_O_4_–S/DPPC monolayer with X_W_ = 0.36, the domains of the nanoparticles with growing size surrounded by the homogenous DPPC film, first in the LE state and then in the LC phase, were visible during the whole compression process. The LC phospholipid domains were not formed (or their size was below the lateral resolution of the BAM microscope used) during the LE–LC phase transition, which explains the absence of the plateau in the *π*–*A* isotherms obtained for Fe_3_O_4_–S/DPPC monolayers with the highest concentrations of nanoparticles.

### 2.3. Dilatational Viscoelasticity

The effect of the Fe_3_O_4_–S and Fe_3_O_4_–AS nanoparticles on the dilatational viscoelasticity of the DPPC monolayer was investigated under dynamic conditions by the oscillating barrier method, which allowed the determination of a dilatational viscoelasticity modulus (*E*) as a complex quantity. The *E* modulus is composed of the real component, elastic modulus, *E*’ and an imaginary component, viscous modulus, *E*”:

*E* = *E*’ + *iE*”. (2)

**Figure 4:**
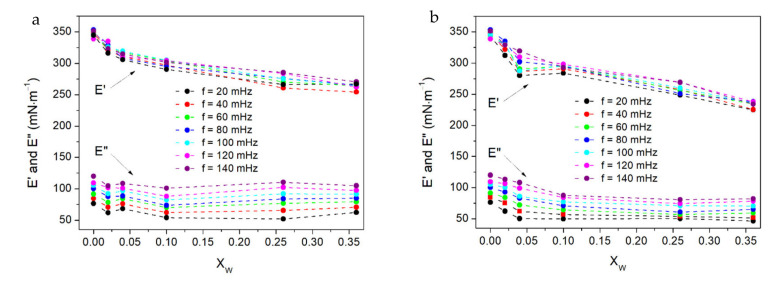
Dependencies of the elastic (*E*’) and viscous (*E*”) modulus of the Fe_3_O_4_–S/DPPC (**a**) and Fe_3_O_4_–AS/DPPC (**b**) films on their composition measured at the different frequencies of the barrier oscillations.

For a perfectly viscous Langmuir film, the real part of *E* is equal to 0, while for an ideally elastic one, the imaginary part takes the value of 0. The surface viscoelastic properties of the Fe_3_O_4_–S/DPPC and Fe_3_O_4_–AS/DPPC films at the air–water interface were investigated at a surface pressure corresponding to that in a native cellular membrane (30 mN·m^−1^) depending on the deformation rate, i.e., the frequency of oscillations (*f*). The *E*’ and *E*” modulus as a function of the composition of the binary films are plotted in Figure 4.

Figure 4 shows that the dilatational elasticity (*E*’) of the Fe_3_O_4_–S/DPPC and Fe_3_O_4_–AS monolayers depends very weakly on the frequency of oscillations in the studied range, while the dilatational viscosity (*E*”) slightly increases with the frequency. In the case of the pure DPPC film, the values of the elastic modulus *E*’ were significantly higher than the values of the viscous modulus *E”*, which indicated the formation of an elastic DPPC monolayer. Thus, although there was a viscoelastic response, the DPPC film exhibited a more elastic character. The presence of the Fe_3_O_4_–S and Fe_3_O_4_–AS nanoparticles did not change the elastic character of the DPPC monolayer. However, the elastic modulus decreased when the concentration of both types of the nanoparticles in the mixed films increased. Simultaneously, the viscous modulus remained almost unchanged in the case of the Fe_3_O_4_–S/DPPC monolayers and dropped slightly in the case of the Fe_3_O_4_–AS/DPPC films. The decrease of *E*’ and thus a reduced elasticity of the binary films was probably caused by the increase in the number and size of the more rigid domains formed by the nanoparticles with their growing concentration. It should be taken into account that the observed effect could also have been caused by the desorption of part of the nanoparticles from the air–water interface during compression of the binary films to the surface pressure of 30 mN·m^−1^, when the oscillatory barrier experiment was performed. The higher concentration of the nanoparticles in the Langmuir monolayer could have led to their stronger desorption.

### 2.4. Adsorption Kinetics

**Figure 5:**
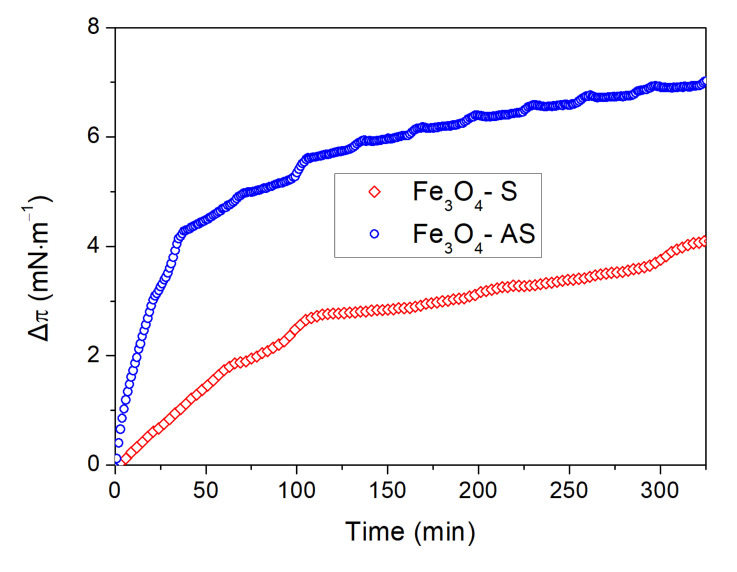
Adsorption kinetics of the Fe_3_O_4_–S and Fe_3_O_4_–AS nanoparticles from the water subphase into the DPPC monolayer recorded at the surface pressure of 30 mN·m^−1^.

To investigate the adsorption of the Fe_3_O_4_–S and Fe_3_O_4_–AS nanoparticles into the DPPC monolayer, the kinetics of this process were recorded at a surface pressure of 30 mN·m^−1^. The change of the surface pressure (Δ*π*) as a function of time was monitored at the fixed barrier position after injection of the suspension of the nanoparticles into the water subphase. In general, the increase in the Δ*π* value above zero indicated the adsorption of the nanoparticles into the phospholipid monolayer. On the other hand, a decrease below zero was related to the extraction of phospholipid molecules from the air–water interface as a result of nanoparticle–phospholipid interactions. The Δ*π* increase was observed after injection into the water subphase of both types of nanoparticles. The Δ*π* took the value of ≈4.2 mN·m^−1^ and 7.1 mN·m^−1^ after 6 h from injection of the Fe_3_O_4_–S and Fe_3_O_4_–AS nanoparticles, respectively, which suggested that they were adsorbed into the DPPC monolayer. Moreover, adsorption was stronger for Fe_3_O_4_–AS nanoparticles (Figure 5).

Free surface energy (γ_S_) and its dispersive (γ_D_) and polar (γ_P_) components were determined for the polymers covering the magnetite core of the nanoparticles (native and aminated starch) to explain their adsorption into the phospholipid monolayer (Table 1). The results clearly indicated a greater hydrophilic nature for the aminated starch surface, its polar component value being almost four times higher than that for native starch. The reason for this greater polarity is the presence of amino groups with an opened pyranose ring as the result of oxidation, which facilitated the alignment of groups at the polymer surface. The hydrophilic “heads” of the phospholipid molecules forming the Langmuir monolayer at the air–water interface were directed towards the water, while their hydrophobic “tails” were turned towards the air. The nanoparticles that adsorbed into the DPPC monolayer during the water subphase first interacted with the “heads” of the DPPC molecules. For this reason, the more hydrophilic Fe_3_O_4_–AS nanoparticles were probably adsorbed more easily to the phospholipid film.

## 3. Materials and Methods

### 3.1. Materials

#### 3.1.1. Synthesis of Native Starch- and Aminated Starch-Coated Magnetite Nanoparticles

Magnetic nanoparticles coated with starch and aminated starch were prepared according to a previously published procedure [44]. Starch-coated magnetite nanoparticles were prepared by in situ co-precipitation from a starch solution at room temperature. To convert starch to aminated starch, the starch shell coated on the surface of the magnetite nanoparticles was first oxidized with sodium periodate (NaIO_4_). Prepared in this way, dialdehyde starch-coated magnetite nanoparticles (Fe_3_O_4_–DAS) were aminated via solvent-free pounding with ethylenediamine to obtain aminated starch-coated magnetic material that was dried by lyophilization for effective water removal.

##### Starch-Coated Magnetite Nanoparticle Synthesis (Fe_3_O_4_–S) 

Starch (1.0 g) was dissolved in water (40 °C) under mechanical stirring. Then iron(II)chloride tetrahydrate (1.85 mmol) and iron(III)chloride hexahydrate (3.75 mmol) were added. After adding dropwise a 30% aqueous solution of NaOH (7.5 mL), a black precipitate formed immediately. It was recovered from the suspension by a magnet, washed with deionized water, and dried by lyophilization.

##### Dialdehyde Starch-Coated Magnetite Nanoparticles (Fe_3_O_4_–DAS) Synthesis

Starch-coated magnetite nanoparticles were dispersed in water, and a solution of sodium periodate (0.7 M, 5 mL) was added to the suspension. The mixture was heated to 40 °C and held under mechanical stirring at this temperature for 1 h. The nanoparticles were isolated after cooling to room temperature by applying a magnet, washed three times with deionized water, and dried under a vacuum (30 °C, 24 h).

##### Aminated Starch-Coated Magnetic Nanoparticles (Fe_3_O_4_–AS) Synthesis

Dialdehyde starch-coated magnetite nanoparticles (0.5 g) were pounded with ethylenediamine (0.02 mL) in an agate mortar at room temperature for 1 min. To remove excess unreacted diamine, washing with water was used until a ninhydrin test showed negative (the amine was not observed in the filtrate). The black solid was dried by lyophilization.

#### 3.1.2. Characterization of the Native Starch- and Aminated Starch Coated-Magnetite Nanoparticles

Characterization of the Fe_3_O_4_–S and Fe_3_O_4_–AS nanoparticles is described elsewhere [44]. A conventional analysis performed in previous studies using transmission electron microscopy showed that the nanoparticles had an average size of 20 nm. According to dynamic light scattering their medium size is equal to 22–25 nm for a particle. X-ray diffraction confirmed that the core of the nanoparticles was made of pure magnetite with a spinel structure. A successful generation of the long-distanced NH_2_ groups on the surface of the Fe_3_O_4_–AS nanoparticles was confirmed by an attenuated total reflectance Fourier transform infrared analysis. The magnetization hysteresis curves recorded for the Fe_3_O_4_–S and Fe_3_O_4_–AS nanoparticles, using superconducting quantum interference, showed a characteristic shape for superparamagnetic materials. The value of the saturation magnetization of the coated nanoparticles was reduced by 46 emu/g compared to the naked ones.

#### 3.1.3. Phospholipids

DPPC was purchased as a powder from Avanti Polar Lipids (Alabaster, AL, USA) and dissolved in spectroscopically pure chloroform (Avantor, Gliwice, Poland) at the concentration of 1 mM to obtain stock solutions.

### 3.2. Methods

#### 3.2.1. Preparation of the Suspensions of the Nanoparticles

Suspensions of the nanoparticles in chloroform and water used in the Langmuir monolayer experiments were prepared according to the following procedure: dispersed in the deionized water from a Milli-Q system (Merck, Darmstadt, Germany) at a concentration of 1 mg·mL^−1^, kept in an ultrasonic washer for 1 h, and filtered through syringe filters having a pore diameter of 200 nm. For filtration of the chloroform suspensions, filters with a Teflon membrane were used, whereas for water suspensions filters with a nylon membrane were applied. The final concentrations of prepared suspensions were measured using a quartz crystal microbalance (openQCM, Pompeii, Italy).

#### 3.2.2. Compression of the Langmuir Films

The binary films formed from the Fe_3_O_4_–S or Fe_3_O_4_–AS nanoparticles and DPPC were compressed using two symmetrically driven hydrophilic barriers on a Langmuir trough (KN 2002, KSV NIMA, Helsinki, Finland) made of Teflon. The through had a surface area of 273 cm^2^ (36.4 × 7.5 cm^2^) and a subphase volume of 176 mL. Before the experiments, the trough was cleaned two times with methanol and chloroform. Ultrapure deionized water with the final resistivity of 18.2 MΩ·cm from the Milli-Q system was used as a subphase. Its constant temperature of 20.0 ± 0.1 °C was kept during experiments by a circulating thermostat F12-ED (Julabo, Seelbach, Germany). The surface of the water subphase filling the Langmuir trough was cleaned using a standalone aspirator pump until the change in surface pressure after maximal slide of the barriers was below 0.1 mN·m^−1^. In the next step, mixtures of a particular weight fraction were prepared from a chloroform suspension of the nanoparticles and a chloroform solution of the phospholipid in a flask. An appropriate amount of a prepared mixture (30–160 μL, depending on the mixture composition) was immediately deposited dropwise onto the clean subphase by a high-precision microsyringe (Hamilton, Reno, NV, USA). Compression of the mixed films took place at the barrier motion speed of 5 mm·min^−1^ after 15 min for chloroform evaporation. During compression, the surface pressure, *π*, was measured using a highly porous platinum Wilhelmy plate hanging from an electronic balance with an accuracy of 0.01 mN·m^−1^ as a function of the mean molecular area per phospholipid molecule *A*. Each *π*–*A* isotherm was recorded at least three times to confirm its reproducibility. The measurements were reproducible within an error of ±0.02 nm^2^.

#### 3.2.3. Brewster Angle Microscopy Observations

Textures of the investigated monolayers were observed in situ by a custom-made Brewster angle microscope (BAM) based on the Moebius set-up [45,46]. The BAM was coupled to the Langmuir trough and set on a table equipped with an active vibration isolation system. A green (532 nm) laser beam was directed at the air–water interface at the Brewster angle of water equal to 53.1°. A CCD camera was used to detect light reflected from the monolayers. A flat black glass plate was placed on the bottom of the Langmuir trough during the experiments to absorb the refracted beam. Only a horizontal stripe in the centre of the recorded BAM images was in focus. A lateral resolution of the used BAM was ≈2 µm.

#### 3.2.4. Oscillatory Barrier Experiment

Dilatational viscoelasticity of the Langmuir films was studied using the oscillating barrier method. This experiment is based on the acquisition of the surface pressure response to the small-amplitude sinusoidal variation of the surface area. The monolayers were first compressed to the surface by a pressure of 30 mN·m^−1^ and allowed to relax for 20 min. Subsequently, the barriers started to oscillate, inducing small amplitude (1%) changes to the area available for the Langmuir films to take place. The experiments were performed for several frequencies from 20 to 140 mHz. At least 10 oscillation cycles were recorded for each frequency. The time interval of 60 s was preserved between subsequent cycles of oscillations.

#### 3.2.5. Penetration Experiment

The adsorption kinetics of the Fe_3_O_4_–S and Fe_3_O_4_–AS nanoparticles into the DPPC monolayer were obtained as a result of penetration experiments. In these experiments, the phospholipid monolayer was compressed on the pure water subphase until the surface pressure reached the value of 30 mN·m^−1^. Then, 5 mL of the water suspension of the Fe_3_O_4_–S or Fe_3_O_4_–AS nanoparticles was injected into the water subphase behind the barriers to avoid disruption of the DPPC monolayer. The concentration of the nanoparticles in the subphase was equal to 3.5 µg·mL^−1^. During the penetration experiment, the surface pressure was monitored at the fixed barrier position as a function of time (*π*^PE^ (*t*)). Simultaneously, the reference experiment was performed, in which the phospholipid film was also compressed to *π* = 30 mN·m^−1^ on the pure water surface. However, the surface pressure (*π*^R^(*t*)) was monitored without injecting the nanoparticle suspension. The change of the surface pressure under the influence of the nanoparticles in the water subphase (Δ*π* (*t*)) was calculated as follows:

Δπ (*t*) = π^PE^ (*t*)− π^R^ (*t*) (3)

The penetration and reference experiments were performed simultaneously to eliminate the effect of water evaporation from the Langmuir trough and any fluctuations of external conditions (temperature or humidity) on the time-consuming experiments.

#### 3.2.6. Surface Free Energy

The hydrophilic/hydrophobic properties of starch- and aminated starch-coated surfaces of the magnetite nanoparticles were analyzed by contact angle measurements using a DSA G10 goniometer (Kruss GmbH, Hamburg, Germany). The image of a sessile drop of glycerin or diiodomethane onto the polymer film surface was recorded and digitized by the camera. The drop-shape analysis and determination of contact angle were done with help of instrument software (Kruss GmbH, Hamburg, Germany). The contact angle value was calculated as an average of at least 10 measurements carried out for each type of polymer and solvent (glycerin or diiodomethane). During measurements, a constant temperature of 23 °C was kept. The surface free energy was calculated by the Owens–Wendt method [47].

## 4. Conclusions

In conclusion, interactions between native starch- and aminated starch-coated magnetite nanoparticles and DPPC molecules in the Langmuir monolayers constituting model cell membranes were studied. The research showed that the nanoparticles with DPPC were able to create stable and compressible binary Langmuir films. However, the *π–A* isotherms recorded during their compression revealed reduced stability and modified phase behavior in comparison to the pure DPPC monolayer. The formation of separate domains by the nanoparticles indicated partial or total immiscibility with DPPC, which was observed under the Brewster angle microscope. The studies on the dilatational viscoelasticity showed that the binary films preserved the elastic character, but their elasticity was reduced in comparison to the DPPC monolayer due to the presence of more rigid nanoparticle domains, the size and number of which grew with nanoparticle concentration. The magnetite nanoparticles coated with native starch disturbed the stability and thermodynamic state of the DPPC monolayer much more than did those functionalized with aminated starch. On the other hand, the kinetics of the adsorption process obtained from the penetration experiments revealed stronger adsorption of the aminated starch-coated nanoparticles into the DPPC film, probably because of more hydrophilic nature. Amino groups attached to the starch in the shell of the magnetite nanoparticles not only made their further functionalization for specific nano–bio applications easier but also seemed to reduce, to some extent, their disruptive effect on the model membranes and improved the adsorption of the nanoparticles. This research, performed on a model simplified cellular membrane, delivered useful information for the future design of magnetite nanoparticles for nanomedicine. However, to fully understand the mechanism of the interactions between nanoparticles and cellular membranes, studies involving bilayers made of different phospholipids under various conditions (pH, temperature) has to be carried out.

## Figures and Tables

**Table 1:** Surface free energy (γ_s_) and its dispersive (γ_d_) and polar (γ_p_) component calculated for the native and aminated starch.

Sample	Surface Free Energy (mJ/m^2^)		
	**γ_s_**	**γ_d_**	**γ_p_**
Starch	34.4	28.8	5.6
Aminated starch	44.1	26.1	18.0

## Data Availability

Not applicable.

## Supplementary Materials

Supplementary materials can be found at https://www.mdpi.com/article/10.3390/ijms22115939/s1.
