# Supplementary material for: Probing of Interactions of Magnetite Nanoparticles Coated with Native and Aminated Starch with a DPPC Model Membrane
Source: Int J Mol Sci. 2021 May 31;22(11):5939. doi: 10.3390/ijms22115939 (PMC8198464; doi:10.3390/ijms22115939)
Supplement: Supplementary file 1 [file ijms-22-05939-s001.zip › ijms-1224573-supplementary.pdf]

SUPPLEMENTARY MATERIALS

Probing of Interactions of the Magnetite Nanoparticles Coated with Native and Aminated Starch with the DPPC Model Membrane

Emilia Piosik [1,*], Aleksandra Zaryczniak [1], Kinga Mylkie [2] and Marta Ziegler-Borowska [2,*]

[1] Faculty of Material Engineering and Technical Physics, Poznan University of Technology, Piotrowo 3, 60-965 Poznan, Poland;
[2] Faculty of Chemistry, Nicolaus Copernicus University in Torun, Gagarina 7, 87-100 Torun, Poland;
* Correspondence: emilia.piosik@put.poznan.pl (E.P.); martaz@umk.pl (M.Z.-B.)

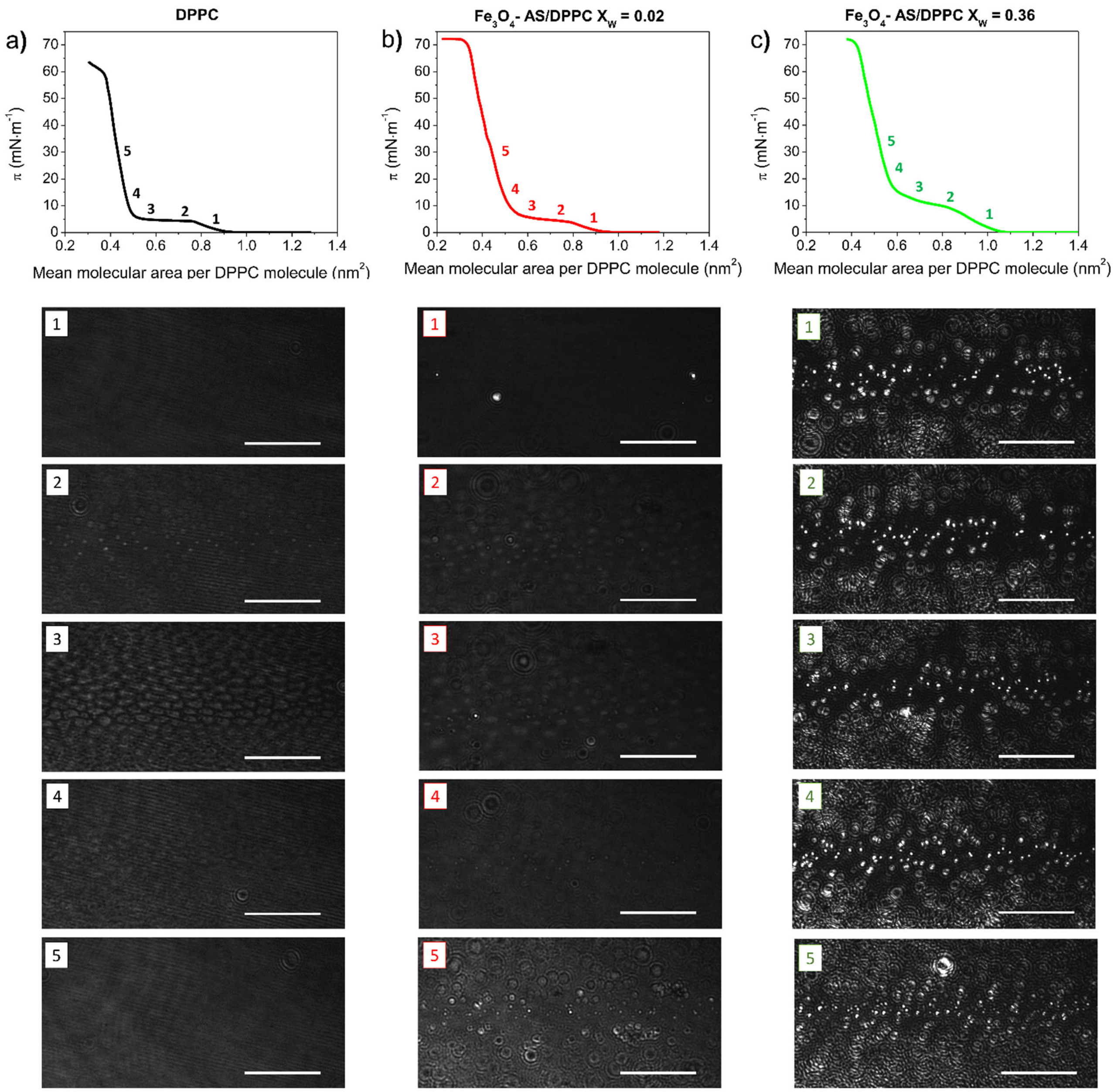

Figure S1. Brewster angle microscope images recorded during compression of the DPPC film (**a**) and Fe_3O_4-AS/DPPC monolayers with X_W=0.02 (**b**) and X_W=0.36 (**c**). Length of scale bar in BAM images—100 µm.
